# Large primary pleural synovial sarcoma with severe dyspnea: a case report

**DOI:** 10.1186/s40792-017-0301-7

**Published:** 2017-02-15

**Authors:** Minoru Yamaki, Shuji Yonehara, Toshio Noriyuki

**Affiliations:** 1 Department of Surgery, Onomichi General Hospital, 1-10-23 Hirahara, Onomichi, Hiroshima 722-8508 Japan; 2 Department of Pathology, Onomichi General Hospital, Onomichi, Hiroshima Japan; 3 Department of Gastroenterological and Transplant Surgery, Applied Life Sciences, Institute of Biomedical and Health Sciences, Hiroshima University, Hiroshima, Japan

**Keywords:** Synovial sarcoma, Pleura, Pleuropneumonectomy, Anterior approach

## Abstract

**Background:**

Synovial sarcoma is a malignant neoplasm of soft tissues. It occurs mainly in the extremities and is closely related to tendons, tendon sheaths, and bursal structures. Primary synovial sarcoma of the pleura and lungs is extremely rare.

**Case presentation:**

We present the case of a 62-year-old man with a large synovial sarcoma of the left pleura. He presented with general fatigue and severe dyspnea. Chest computed tomography (CT) revealed a 20-cm tumor in the left thoracic cavity. We first diagnosed the tumor as a sarcomatoid mesothelioma based on CT-guided needle biopsy. We speculated that his severe dyspnea was because of ventilation-perfusion mismatch due to the left pulmonary collapse. Furthermore, we thought that there was a discrepancy between the CT findings and the pathological findings from the biopsy specimen. We performed pleuropneumonectomy through an anterior approach with median sternotomy and 5th-intercostal thoracotomy. The resected specimen contained a 22-cm pleural tumor with parenchymatous hemorrhage. We diagnosed the tumor as monophasic synovial sarcoma based on its morphologic and immunohistochemical features. We suspected there was microscopic residual tumor in the left diaphragm and therefore performed radiation therapy. After radiotherapy, he received adjuvant chemotherapy with ifosfamide and Adriamycin. One year after surgery, the patient is alive with no signs of tumor recurrence.

**Conclusions:**

We report a case of a large synovial sarcoma of the pleura in a patient with severe dyspnea. He was treated with pleuropneumonectomy, radiotherapy, and adjuvant chemotherapy. Although the best treatment for this rare condition has not been defined, we thought that tumor resection and adjuvant therapy were appropriate to control the disease in this case.

## Background

Primary synovial sarcoma of the pleura is extremely rare. Optimal treatment for synovial sarcoma of the pleural has not been defined. We describe a case of large synovial sarcoma of the pleura, which was resected via pleuropneumonectomy.

## Case presentation

**Fig. 1:**
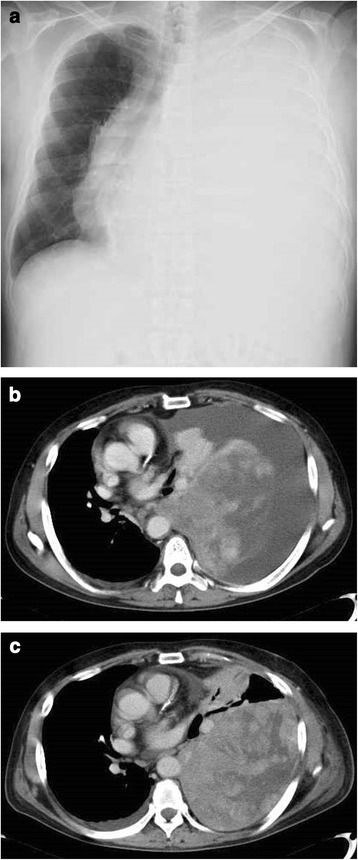
Preoperative imaging studies. The chest radiogram shows a large pleural effusion in the left thoracic cavity and a mediastinum shift to the right (**a**). Contrast-enhanced computed tomography (CT) on admission shows a 20-cm tumor and pleural effusion in the left thoracic cavity (**b**). Contrast-enhanced CT a month after admission shows that the tumor has rapidly grown and the right pleural effusion has increased since admission (**c**)

A 62-year-old man presented with general fatigue and severe dyspnea. He was an ex-smoker. He did not have a history of exposure to asbestos. Physical examination revealed oxygen saturation of 90% (room air) and decreased left vesicular sounds. Arterial blood gas analysis indicated the following results: pH 7.4752, pO_2_ 73.9 mmHg, and pCO_2_ 24.8 mmHg (O_2_ 2 L/min). Spirometry was not performed because of severe dyspnea. Chest radiography showed a large pleural effusion in the left thoracic cavity (Fig. 1a). On chest computed tomography (CT), a 20-cm tumor in the left thoracic cavity was visible (Fig. 1b). Removal of pleural fluid was performed, and the cytology of the pleural fluid was negative for malignancy. CT-guided needle biopsy was performed. The pathological findings showed a proliferation of atypical short spindle or oval cells with hyperchromatic nuclei and eosinophilic cytoplasm. Immunohistochemically, the tumor cells were positive for D2-40, BCL-2, CD99, and p53; they were negative for TTF-1, WT1, calretinin, cytokeratin (CK)5/6, CK7, CK20, HBME-1, CEA, and CD34. The biopsy specimen was diagnosed as a sarcomatoid mesothelioma. One month after admission, CT showed that the tumor had rapidly grown in size and the pleural effusion in the right cavity was slightly increased (Fig. 1c). We thought that the pleural effusion in the right cavity was due to inflammation. We did not perform thoracentesis for the right pleural effusion. We speculated that his severe dyspnea was because of ventilation-perfusion mismatch caused by the left pulmonary collapse. Furthermore, we thought that there was a discrepancy between CT findings and pathological findings from the biopsy specimen. We planned a pleuropneumonectomy to improve of his performance status. The tumor was too large to be removed using the usual posterolateral incision; hence, we performed an anterior approach with median sternotomy and 5th-intercostal thoracotomy. Because the adhesion between the tumor and diaphragm was tight, it was difficult to perform en bloc resection of the tumor. We performed partial resection of the diaphragm which was adhered with tumor tightly. Although the tumor was completely resected macroscopically, we suspected there was microscopic residual tumor in the left diaphragm. The surgical operating time was 306 min, and total blood loss was 3180 cc. His postoperative course was uneventful, and he was discharged on postoperative day 21. His performance status had improved from 3 to 1.

**Fig. 2:**
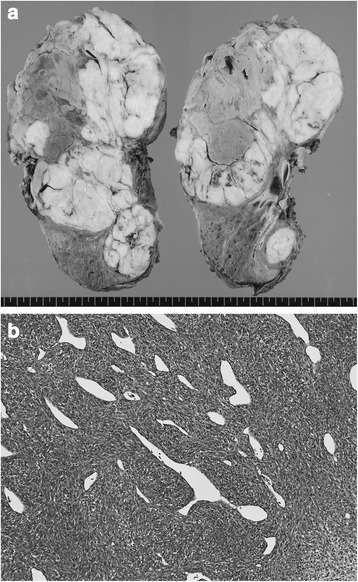
Pathological examination. The cut surface of the resected specimens shows a pleural tumor with parenchymatous hemorrhage (**a**). Microscopic examination (hematoxylin-eosin staining) shows a proliferation of atypical short spindle or oval cells with hyperchromatic nuclei and eosinophilic cytoplasm arranged in fascicles, whorls, or in a herringbone pattern with necrotic foci, associated with gaping thin-walled blood vessels displaying a hemangiopericytoma-like appearance (**b**)

The resected specimen showed a 22-cm pleural tumor with parenchymatous hemorrhage (Fig. 2a). Pathological findings showed a proliferation of atypical short spindle or oval cells with hyperchromatic nuclei and eosinophilic cytoplasm arranged in fascicles, whorls, or in a herringbone pattern with necrotic foci, associated with gaping thin-walled blood vessels displaying a hemangiopericytoma-like appearance (Fig. 2b). Mitotic figures were readily encountered.

Immunohistochemically, the tumor cells were positive for epithelial membrane antigen (EMA), D2-40, BCL-2, CD99, and p53. They were negative for TTF-1, WT1, calretinin, cytokeratin (CK)5/6, CK7, CK20, HBME-1, CEA, and CD34. Because the tumor cells were positive for CD99 and BCL-2, which are usually negative in a case of malignant mesothelioma, malignant sarcomatoid mesothelioma seemed to be less of a possibility. In a molecular assay using the formalin-fixed, paraffin-embedded tumor tissue, no *SS18-SSX* fusion gene transcript, a characteristic of synovial sarcoma, was identified despite the detection of messages of housekeeping genes (*PGK* and *PBGD*) used as internal controls. In addition, the fluorescence in situ hybridization (FISH) analysis using an *SS18* break apart probe failed to identify a convincing gene rearrangement. However, synovial sarcoma cannot be ruled out because it can have a minor fusion gene variant (*SS1821-SSX*), which we did not test for.

We diagnosed the tumor as a monophasic synovial sarcoma based on its morphologic and immunohistochemical features. Because we suspected that there was residual microscopic tumor, 50 Gy of radiation was delivered to the diaphragm. Thereafter, adjuvant chemotherapy with ifosfamide and Adriamycin, which are administered for cases of soft tissue sarcoma in the extremities, was administered. Currently, he is alive and doing well, without evidence of recurrent disease 12 months after the surgery.

Synovial sarcoma is a malignant neoplasm of soft tissues. It occurs mainly in the extremities and is closely related to tendons, tendon sheaths, and bursal structures. Primary synovial sarcoma of the pleura and lungs is extremely rare [1–3]. The common histological subtypes of synovial sarcoma are biphasic, monophasic spindle cell or fibrous, monophasic epithelial, and poorly differentiated subtypes [4]. The biphasic type is easily diagnosed based on the presence of both epithelial and spindle cell components. The monophasic type is difficult to diagnose, because it has a uniform spindle cell pattern, which is similar to other malignant spindle cell neoplasms [5].

In our case, at first, we diagnosed the tumor as a sarcomatoid mesothelioma. In the biopsy specimen, we did not find a proliferation of atypical cells with a hemangiopericytoma-like appearance, which was found in the resected specimen. Immunohistochemical findings play an important role in the differential diagnosis of synovial sarcoma. Synovial sarcoma is almost always positive for cytokeratins and EMA. Synovial sarcoma lacks staining for neural (S100) and smooth muscle (desmin and smooth muscle actin) markers [6]. The chromosomal translocation t(X;18)(p11.2;q11.2) has been found in more than 90% of synovial sarcomas, regardless of histologic subtype [7]. This translocation results in the fusion of the *SYT* gene on chromosome 18 to either the *SSX1* or *SSX2* gene on chromosome X [8]. In our case, no *SS18-SSX* fusion gene transcript was identified. In addition, the FISH analysis using an *SS18* break apart probe failed to identify a convincing gene rearrangement. However, the tumor was diagnosed as monophasic synovial sarcoma based on its morphologic and immunohistochemical features.

Optimal treatment for synovial sarcoma of pleura has not been defined. Multimodal therapy involving surgery, chemotherapy, and radiotherapy has been used. Radical resection with an adequately wide margin is the standard operation, similar to that used for other soft tissue sarcomas [9]. Adjuvant radiotherapy is usually recommended after incomplete resection. Adjuvant chemotherapy using doxorubicin ± ifosfamide is significantly beneficial in terms of 5-year disease-free and overall survival in cases of soft tissue sarcoma [10]. In a phase II study of perioperative chemotherapy with ifosfamide and doxorubicin for high-grade soft tissue sarcomas in the extremities including synovial sarcoma, the 2- and 5-year progression-free survival rates were found to be 75.7% (95% CI, 63.9–84.1%) and 63.8% (95% CI, 51.3–73.9%), respectively, and the 5-year overall survival was 82.6% (95% CI, 71.3–89.7%) [11].

In our case, microscopically residual tumor in the left diaphragm was suspected, and therefore, radiation therapy was performed. After radiotherapy, adjuvant chemotherapy with ifosfamide and Adriamycin, which are administered for soft tissue sarcoma in the extremities, was administered. Galetta reported 15 cases of primary thoracic synovial sarcoma. Factors that adversely affect survival include a tumor dimension of greater than 10 cm, incomplete resection, and no adjuvant therapy [12].

In our case, we initially diagnosed a sarcomatoid mesothelioma based on CT-guided needle biopsy. The preoperative TNM classification for malignant pleural mesothelioma according to the International Mesothelioma Interest Group was stage II (T2N0M0). The National Comprehensive Cancer Network Guidelines for malignant pleural mesothelioma recommend chemotherapy for sarcomatoid mesothelioma. Indications for surgery in our case were controversial. In our case, the large tumor was causing ventilation-perfusion mismatch through the left pulmonary collapse. Therefore, the patient experienced severe dyspnea. The tumor rapidly grew over a 1-month period. The patient was relatively young and had no comorbidities. We thought that resection of the large tumor would improve his condition. Thus, this was a rescue surgical intervention case for severe dyspnea and a critical status. Furthermore, we thought that there was a discrepancy between the CT findings and the pathological findings of the biopsy specimen, which was an additional reason for performing surgical resection. After surgery, the patient’s respiratory condition improved and his performance status improved from 3 to 1. Hara reported a similar case with very rapid progression of the tumor, and the patient died only 2 months after first presentation [13]. We thought that tumor resection for control of the disease was appropriate.

## Conclusions

We report a case of a large synovial sarcoma of the pleura in a patient with severe dyspnea, who was treated with pleuropneumonectomy, radiotherapy, and adjuvant chemotherapy. Although the best treatment has not been defined, we thought that tumor resection and adjuvant therapy were appropriate for control of the disease in this case.

## Abbreviations

CK: Cytokeratin
CT: Computed tomography
EMA: Epithelial membrane antigen
FISH: Fluorescence in situ hybridization
